# Social Engagement and Urban–Rural Disparity in Self-Management Behaviors: Study of Middle-Aged and Older Chinese Hypertension Patients

**DOI:** 10.3389/fpubh.2021.801307

**Published:** 2022-01-27

**Authors:** Jiao Lu, Linhui Liu, Yuan Wang, Zhongliang Zhou

**Affiliations:** ^1^School of Public Policy and Administration, Xi'an Jiaotong University, Xi'an, China; ^2^School of Management, Shanxi Medical University, Taiyuan, China

**Keywords:** urban/rural, self-management behaviors, social engagement, hypertension, China

## Abstract

**Background:**

This study examines the effect of social engagement on urban–rural disparities in self-management behaviors (medication use, self-monitoring, physical activity, and tobacco and alcohol avoidance) among middle-aged and older Chinese patients with hypertension.

**Methods:**

Data from 2011 to 2018 were extracted from the four latest waves of the China Health and Retirement Longitudinal Study. Chi-square tests and *t*-tests were performed to examine urban–rural gaps in self-management behaviors. Random-effects panel logit regression models were adopted to confirm the effect of social engagement on urban–rural disparity in self-management behaviors and to explore whether this effect has narrowed or widened with “bilateral flow” between urban and rural residents. A Fairlie decomposition technique was also used to calculate the extent to which social engagement reflects urban–rural disparities.

**Results:**

There was significant urban–rural disparity in medication use and tobacco avoidance behaviors among the sampled patients. Medication use behavior (*p* < 0.001) among urban middle-aged and older patients was significantly better, whereas tobacco avoidance behavior (*p* < 0.05) was significantly lower compared with the rural population. Social engagement significantly enlarged the urban–rural gap in tobacco avoidance behavior (*p* < 0.01), but significantly narrowed the urban–rural gap in medication use behavior (*p* < 0.001). The Fairlie decomposition revealed that ~75.000% and 29.412% of the explained urban–rural gap in tobacco avoidance and medication use, respectively, could be attributed to social engagement. The negative effect of social engagement on urban–rural disparity in medication use increased when urban residents moved to rural areas (*p* < 0.05).

**Conclusions:**

The urban–rural disparities in self-management behaviors of middle-aged and older Chinese hypertensive patients were mainly manifested in medication use and tobacco avoidance behaviors. The gaps in these two behaviors partly changed with social engagement, while the migration of urban population to rural areas weakens the positive role of social engagement in narrowing the urban–rural gap in medication use behavior. The insights of this paper on social engagement and urban–rural disparity in self-management behaviors, and the effect of urban–rural migration thereof, deserve the attention of health policymakers and researchers.

## Introduction

Self-management, as a type of self-care, prioritizes prevention and care for patients with chronic disease; and have stressed by healthcare professionals in China (1). Especially with the continuous prevalence of chronic diseases, its important role in disease control and medical resource saving is increasingly prominent (2). Intrinsically, self-management recognizes and seeks to foster patients' daily responsibility to maintain their well-being, by supporting from internal (e.g., family or friend support) and community resources (3). But, owing to the differences between China's urban and rural economic development and the household registration system design, there are wide variations in people's access to life opportunities and social service resources (e.g., healthcare and education) (4, 5). This longstanding urban–rural disparity seems to mean that rural residents are less able to obtain valuable healthcare resources (e.g., correct health information and diversified healthcare services) to improve and maintain their self-management behaviors compared with urban residents (6).

Undoubtly, the available healthcare resources are not equal to the actual healthcare resources utilized by individuals. As both Bourdieu and Veenstra in their work on social capital theory note that rich healthcare resources only mean an increasing chances and opportunities to access resources (7, 8); the maximum utilization of healthcare resources is closely related to the individuals' ability to maintain long-term social connections and participation in social real-life activities (9–11), which is social engagement. This because that effective self-management demands social interaction and collaboration between patients and their environment so they may successfully manage the difficulties that arise from having a chronic illness (12–15).

Correspondingly, the dual-track system between urban and rural areas in Chinese society has led to the dual development of social nature and relationships (16). As a result, there are differences in the characteristic and level of social engagement between urban and rural, which become the main reason for the urban-rural disparity in patients' self-management. Specifically, rural society is more similar to an acquaintance society, with low-level marketization or modernization, while urban society has gradually become a stranger society, with high-level marketization or modernization (17, 18); the former increases more general trust and connections among people than the latter, which may have a positive contextual effect on engagement in social activities (19). Activity theory posits that those who engage in social activities and intimate peer relationships are more likely to maintain a positive adjustment for their illness (11, 20, 21), and rural patients may benefit from the encouragement of group members and a pleasant atmosphere by their peer group to further contribute to a continued self-management (22). Conversely, as Berkman and Glass's (2000) social network theory claims that the high-level general trust contexts are not entirely beneficial for rural people. This context may be more occluded and hinders their access to new health information and ability to engage in more meaningful social activities (e.g., misperceptions of disease management advice owing to a peer's wrong cognition or being forced to smoke and drink during their social interaction) (23–25). So, this eventually leads to unhealthy self-management behaviors (19). While for the urban patients, they are better educated and have enough cognition and opportunity to participate in activities who are often purposeful—although more or less constrained—in expressing and supporting a valued current state or in working toward a desirable goal. Such meaningful engagement offers a source of motivation (e.g., peer pressure and positive role models) and capability (e.g., acquire and identify valuable information) for more healthy behaviors (22, 26). When social engagement involves valued activities that reflect people's needs and interests, it fosters their activeness and sense of control/self-efficacy in practicing self-management behaviors (15). Overall, the negative aspects of social engagement are independent of their positive influences (27), these together form an unknown influence on patients' self-management behaviors, then further impact the urban-rural disparity in self-management.

Furthermore, the disparities discussed may become more complicated when the subjects are middle and old age groups suffering from chronic illness; especially in China, this scenario is further aggravated by frequent “bilateral flow” between urban and rural areas owing to the rapid new urbanization of 2014 and the rural revitalization strategy of 2017 (28). Urbanization has brought about massive internal migration of the labor population from the rural to the urban, and enlarged the geographical separation between migrated adult children and their rural “left-behind” parents or children. Such rural “left-behind” parents have to care for their grandchildren, which reduces their engagement in social activities. Even those who move to urban areas with their adult children have difficulty accessing healthcare resources (29–31). This because the elimination of hukou system alone has not addressed inequities in access to social services between rural and urban populations (32), despite significant reforms to the system to allow rural migrants easier access to welfare programs in cities (31). China implements the household registration system before 1984 completely restricted the movement of labor between rural and urban, which made these middle and old patients migrator form the thoughts and habits in their youth in out-migrating places, leading they are unable to meaningfully integrate into the social milieu of in-migrating places owing to a lack of family and friends; they also lose their prior access to social activities of out-migrating places.

The literature does seem to suggest that social engagement has potentially positive influences on patients' self-management (33–36), and it may also be a determinate factor thereof in the Chinese context. Also, the literatures point out that patients' social engagement change with their social environment live in (37), and it is necessary to design different engagement programs for different groups (38, 39). But little has been researched on understanding the underlying effect of social engagement on urban–rural disparities in self-management behaviors, and this may be detrimental to the designing of interventions intended to maximize the potential of social engagement.

With all that said, chronic diseases are difficult to cure and increasing complicatedly with age. The long-term coexistence of patients and diseases causes serious damage to their physical and psychological health. By clarifying the effect of social engagement on the urban-rural disparity in self-management behaviors, and this effect changes with the population mobility, which is helpful for healthcare workers to develop targeted social engagement strategies to improve the self-management awareness of urban and rural patients, narrow the urban-rural disparity in population health, balance the development of urban and rural areas and promote the effective integration of migrant population further. This is not only a much favorable way to promote the health of middle and old age patients with chronic diseases, but also one of the most valuable measures to help avoid excessive waste of medical and healthcare resources in China, where “getting old before getting rich” and the burden of chronic diseases is increasingly severe.

Thus, to inform future strategies of improving the well-being of middle-aged and older adults living with a chronic condition, this study aims to better understand the urban–rural disparity in chronic illness self-management behaviors and to identify how this disparity is shaped by the social activities in which patients are engaged. What's more is that chronic diseases are too numerous to be studied one by one. Given the prevalence of hypertension in China and the importance of self-management in controlling this illness, we restrict our subsample to hypertensive patients for three reasons. First, according to the Report on cardiovascular diseases in China 2018 and 2019 Chinese guideline for the management of hypertension in the elderly (40, 41), there are about 0.245 billion patients suffering from hypertension in China, the prevalence of hypertension in the oldest old is close to 90%, and these prevalence rates are increasing year by year. Second, since hypertension is a pre-basic disease of a variety of chronic diseases (e.g., stroke, kidney disease, Alzheimer's disease, and neurological disease, etc.), hypertension management has become a part of Essential Public Health Services (EPHS) in China. Third, with the popularity of instruments such as blood pressure monitors, self-management of hypertension is relatively easier to achieve than other diseases.

## Materials and Methods

### Data

Our primary database is drawn from the 2011, 2013, 2015, and 2018 waves of the China Health and Retirement Longitudinal Study (CHARLS). CHARLS is a nationally representative longitudinal survey of persons 45 years of age or older and their spouses, and it includes the social, economic, and health assessments of community residents. The survey adopts multi-stage stratified probability proportionate to size sampling and includes about 10,000 households and 17,500 individuals in 150 counties/districts and 450 villages/resident committees (or villages) from 28 provinces; it uses a face-to-face computer-assisted personal interview.

We are interested in exploring the relationship between the self-management behaviors and social engagement of hypertensive patients in middle and old age over or equal to 45 years old. A total of 15,271 middle-aged and older hypertensive patients were drawn from the 2011, 2013, 2015, and 2018 waves of the CHARLS. We eliminated 869 samples with missing values on social engagement and 807 samples with missing data on self-management behavior. Then we excluded 678 samples with physical disability and 421 samples with cognitive disability. A final sample of 12,516 participants was obtained, 45 years of age or older, split between 9,242 with rural hukou and 3,274 with urban hukou.

### Measures/Variables

The main dependent variables of this study were hypertensive middle-aged and older patients' self-management behaviors, namely, alcohol avoidance, tobacco avoidance, medication use, physical activity, and self-monitoring behaviors (Table 1). Tobacco avoidance behavior was evaluated by two questions, “Have you ever chewed tobacco, smoked a pipe, smoked self-rolled cigarettes, or smoked cigarettes/cigars?” and “Do you still have the habit or have you totally quit?” with “quit or never smoking” scored at 1, and 0 for “stilling smoking.” Alcohol avoidance behavior was evaluated by the question “Did you drink any alcoholic beverages, such as beer, wine, or liquor in the past year? How often?” with “never drinking,” that is, alcohol avoidance, recorded as 1, and “having drunk” as 0. Medication use status was assessed by the question “Are you now taking any of the following medications to treat or control your hypertension?” with use of Chinese traditional or modern medicine to treat and control hypertension recorded as 1, and 0 otherwise. Physical activity was classified into two categories according to CHARLS, namely, intensity and amount of exercise and exercise time, with a score of 1 for participating in regular physical exercise (e.g., ordinary walking or moderate intensity physical activity for not <30 min each time less than three times a week), 0 otherwise. Self-monitoring behavior was assessed by the number of blood pressure examinations in a year, self-reported by the respondents, with the response of one or more times recorded as 1, and 0 otherwise.

**Table 1:** Variable definitions.

**Variables**	**Description**
Alcohol avoidance	1 = Quit or never smoking, 0 = Stilling smoking
Tobacco avoidance	1 = Never drinking, 0 = Having drunk
Medication use	1 = Use of Chinese traditional or modern medicine to treat and control hypertension, 0 = Otherwise
Physical activity	1 = Participating in regular physical exercise = 1, 0 = Otherwise
Self-monitoring	1 = One or more times of blood pressure examinations, 0 = Otherwise
Social engagement	Continuous variable
**Control variables**	
Gender	1 = Female, 0 = male
Age	Continuous variable
Education	1 = Junior school and above, 0 = Primary school and below
Spouse	1 = Yes, 0 = No
Residence	1 = Residence inconsistent with the hukou type, 0 = Otherwise
Absolute household income	Continuous variable
Basic medical insurance	1 = Yes, 0 = No
Self-care ability	Continuous variable
Multimorbidity	1 = Yes, 0 = No

The key independent variables were whether a respondent belongs to an urban or rural registration (hukou dummy) and social engagement. The hukou variable was measured by the question “Which type of household registration do you belong to?” The information on social engagement was obtained by two questions derived from CHARLS. The participants were first asked whether they had participated in any social activities in the past month^1^. Next, they were asked about the frequency of activity in the past month, and the responses were scored on a three-point scale, ranging from 3 (almost every day) and 2 (almost every week) to 1 (not often). The total score of social engagement was 0–33, with higher scores indicating high level of social engagement.

Based on the literature (13, 22, 26), potentially related covariates were classified into sociodemographic characteristics, socio-economic characteristics, and health condition. The individual's sociodemographic characteristics were collected based on the following variables: gender (male = 0, female = 1), age (continuous variable), spouse (yes = 1, no = 0), and residence (residence inconsistent with the hukou type = 1, otherwise = 0). The socio-economic status of the patients included education attainment (junior high school and above education was coded as 1), absolute household income (continuous variable; absolute income was computed based on information on several economic activity domains that were collected from household heads), and whether the respondent had basic medical insurance (yes = 1, no = 0). Health condition included self-care ability and multimorbidity status; the former (continuous variable) was assessed by activities of daily living, where the functional status included assessing six daily tasks, namely, dressing, eating, using the toilet, bathing, indoor activities, and continence. Each item was independently scored with one point, or 0 otherwise, with a 0–6 score range. Multimorbidity status was determined by whether an individual had other chronic diseases besides hypertension (yes = 1, no = 0)^2^.

### Statistical Analyses

The data were analyzed by Stata 15.0 software and processed by descriptive statistics, random-effects panel logit regression, and Fairlie decomposition. We conducted a Chi-square test and *t*-test to examine whether there were statistically significant differences between urban and rural middle-aged and older hypertensive patients. We, thus, adopted a random-effects panel logit regression to investigate the urban–rural disparity in self-management behaviors, the role of social engagement in self-management behaviors, and its effect on the urban–rural disparity in self-management behaviors adjusted for confounding variables. The threshold for statistical significance was set at *p* < 0.05.

First, to analyze the urban–rural disparity in self-management behaviors and the role of social engagement thereof, we constructed a benchmark random-effects panel logit model based on Wu et al. (42):


(1) $$\begin{array}{l} Alc_{it}/Cig_{it}/Med_{it}/Phy_{it}/Moni_{it}\;=\;\beta_{0}+\beta_{1}Enga_{it}\\ \;+\beta_{2}Hukou{\left(urban\right)}_{it}+\gamma Control_{it}+\varepsilon_{i} \end{array}$$

In Model 1, *Alc*_*i*_, *Cig*_*i*_, *Med*_*i*_,*Phy*_*i*_, and *Moni*_*i*_ are respondent *i*'s self-management behaviors, alcohol avoidance, tobacco avoidance, medication use, physical activity, and self-monitoring behaviors, respectively; *Hukou*(*urban*) denotes whether respondent *i*'s hukou was urban or rural; *Enga* is respondent *i*'s level of social engagement; *t* denotes the years 2011, 2013, 2015, and 2018; β_0_ is the constant term; β_1_ and β_2_ are the estimated parameters of social engagement and *Hukou*(*urban*), respectively; *Control*_*i*_ is the control variable; and ε_*i*_ is the random error term.

Second, to verify the effect of social engagement on urban–rural disparity in self-management behaviors, the interaction item of hukou dummy and social engagement was added based on Model 1:


(2) $$\begin{array}{l} Alc_{it}/Cig_{it}/Med_{it}/Phy_{it}/Moni_{it}=\beta_{0}+\beta_{1}Enga_{it}\\ +\beta_{2}Hukou{\left(urban\right)}_{it}+\beta_{3}(Hukou{\left(urban\right)}_{it}*Enga_{it})\\ \;+\gamma Control_{it}+\varepsilon_{i} \end{array}$$

In Model 2, β_3_ is the parameter of the interaction item of hukou dummy and social engagement, and the interpretation of other indicators was consistent with Model 1.

Third, to further study the effect of population mobility on urban–rural differences in self-management behavior caused by social engagement, the interaction item of hukou dummy, social engagement, and residence was added based on Model 2:


(3) $$\begin{array}{l} Alc_{it}/Cig_{it}/Med_{it}/Phy_{it}/Moni_{it}\;=\;\beta_{0}+\beta_{1}Enga_{it}\\ +\beta_{2}Hukou{\left(urban\right)}_{it}+\beta_{3}(Hukou{\left(urban\right)}_{it}*Enga_{it})\\ \;+\beta_{4}(Resi_{it})+\beta_{5}(Hukou{\left(urban\right)}_{it}*Enga_{it}*Resi_{it})\\ \;+\gamma Control_{it}+\varepsilon_{i} \end{array}$$

In Model 3, β_4_ is the parameter of the residence; β_5_ is the parameter of the interaction item of hukou dummy, social engagement, and residence; and the interpretation of other indicators was consistent with Model 1.

After determining the effect of social engagement on the urban–rural disparity in self-management behaviors, we used the Fairlie decomposition method to estimate the contribution of social engagement to the explained difference of urban–rural disparity in self-management behaviors (43). For the Fairlie decomposition technique—a counterfactual method—we assumed what the self-management behavior would be if the rural hukou patients had the same social engagement characteristics as the urban hukou patients. We, thus, used the Fairlie non-linear decomposition method to decompose the urban–rural difference of self-management behaviors into two respective parts:


(4) $$\begin{array}{l} {\bar{Y}}^{U}-{\bar{Y}}^{R}=\left[\sum_{i=1}^{N^{U}}\frac{F\left(X_{i}{\;}^{U}\beta^{U}\right)}{N^{U}}-\sum_{i=1}^{N^{R}}\frac{F\left(X_{i}{\;}^{R}\beta^{U}\right)}{N^{R}}\right]\\ \;+\left[\sum_{i=1}^{N^{R}}\frac{F\left(X_{i}{\;}^{R}\beta^{U}\right)}{N^{R}}-\sum_{i=1}^{N^{R}}\frac{F\left(X_{i}{\;}^{R}\beta^{R}\right)}{N^{R}}\right] \end{array}$$

In Model 4, ${\bar{Y}}^{U}$ and ${\bar{Y}}^{R}$ are the average values of the self-management behaviors of patients with urban hukou and rural hukou, respectively. The superscript “*U*” denotes patients with urban hukou, and the superscript “*R*” represents patients with rural hukou; *N*^*U*^ and *N*^*R*^ are the sample size of the patients with urban hukou and rural hukou, respectively; $X_{i}^{U}$ and $X_{i}^{R}$ are explanatory variables for urban and rural samples; β is the estimated coefficient.

In addition, the first term of Model 4 is the explained component (or endowments effect), that is, the difference caused by the social engagement characteristics of different hukou. The second term represents the unexplainable component (or coefficient effect) caused by unobservable factors. The unexplainable component persists even if the disadvantaged group were to attain the same average levels of measured variables as the advantaged group. Moreover, the variables are randomly ranked 100 times to avoid the influence of the ranking effect of variables on the decomposition results in the Fairlie non-linear decomposition models.

## Results

### Descriptive Results

Table 2 presents the descriptive statistics of the variables used in this study for the rural and urban samples. There were no significant differences in alcohol avoidance, physical activity, and self-monitoring behaviors between either population. Rural respondents avoided tobacco significantly more than urban respondents (*p* < 0.05), but their proportion of medication use behavior was significantly lower in comparison (*p* < 0.05). Their social engagement was also significantly lower (*p* < 0.05).

**Table 2:** Descriptive statistics.

**Variables**	**Rural**	**Urban**	**All**	***p* ^a^**
Alcohol avoidance, *n* (%)				0.93
No	5218 (56.46)	1851 (56.54)	7069 (56.48)	
Yes	4024 (43.54)	1423 (43.46)	5447 (43.52)	
Tobacco avoidance, *n* (%)				0.02
No	4942 (53.47)	1828 (55.83)	6770 (54.09)	
Yes	4300 (46.53)	1446 (44.17)	5760 (45.91)	
Medication use, *n* (%)				0.00
No	1973 (21.35)	520 (15.88)	2493 (19.92)	
Yes	7269 (78.65)	2754 (84.12)	10023 (80.08)	
Physical activity, *n* (%)				0.08
No	5198 (56.24)	1784 (54.49)	6982 (55.78)	
Yes	4044 (43.76)	1490 (45.51)	5534 (44.22)	
Self-monitoring, *n* (%)				0.07
No	2136 (23.11)	706 (21.56)	2842 (22.71)	
Yes	7106 (76.89)	2568 (78.44)	9674 (77.29)	
Social engagement (SD)	4.35 (4.67)	5.96 (5.64)	4.77 (4.99)	0.00
**Control variables**				
Gender, *n* (%)				0.00
Male	4075 (44.09)	1740 (53.15)	5815 (46.46)	
Female	5167 (55.91)	1534 (46.85)	6701 (53.54)	
Age (SD)	62.85 (9.73)	64.11 (9.98)	63.18 (9.81)	0.00
Education, *n* (%)				0.00
Primary school and below	3675 (39.76)	637 (19.46)	4312 (34.45)	
Junior school and above	5567 (60.24)	2637 (82.54)	8204 (65.55)	
Spouse, *n* (%)				0.15
No	1536 (16.62)	509 (15.55)	2045 (16.34)	
Yes	7706 (83.38)	2765 (84.45)	10471 (83.66)	
Residence, *n* (%)				0.00
Rural	8583 (92.87)	984 (30.05)	9567 (76.44)	
Urban	659 (7.13)	2290 (69.95)	2949 (23.56)	
Absolute household income (SD)	695.95 (1937.69)	1372.01 (3231.42)	872.79 (2364.65)	0.00
Basic medical insurance, *n* (%)				0.77
No	312 (3.38)	114 (3.48)	426 (3.40)	
Yes	8930 (96.62)	3160 (96.52)	12090 (96.60)	
Self-care ability (SD)	9.21 (2.85)	9.06 (2.94)	9.1684 (2.87)	0.01
Multimorbidity, *n* (%)				0.00
No	2234 (24.17)	648 (19.79)	2882 (23.03)	
Yes	7008 (75.83)	2626 (80.21)	9634 (76.97)	
Wave, *n* (%)				0.00
2011	2728 (29.52)	973 (29.72)	3701 (29.57)	
2013	1783 (19.29)	760 (23.21)	2543 (20.32)	
2015	3032 (32.18)	1006 (30.73)	4038 (32.26)	
2018	1699 (18.38)	535 (16.34)	2234 (17.85)	

a *χ^2^tests for dichotomous variables and t-tests for continuous variables*.

**Figure 1:**
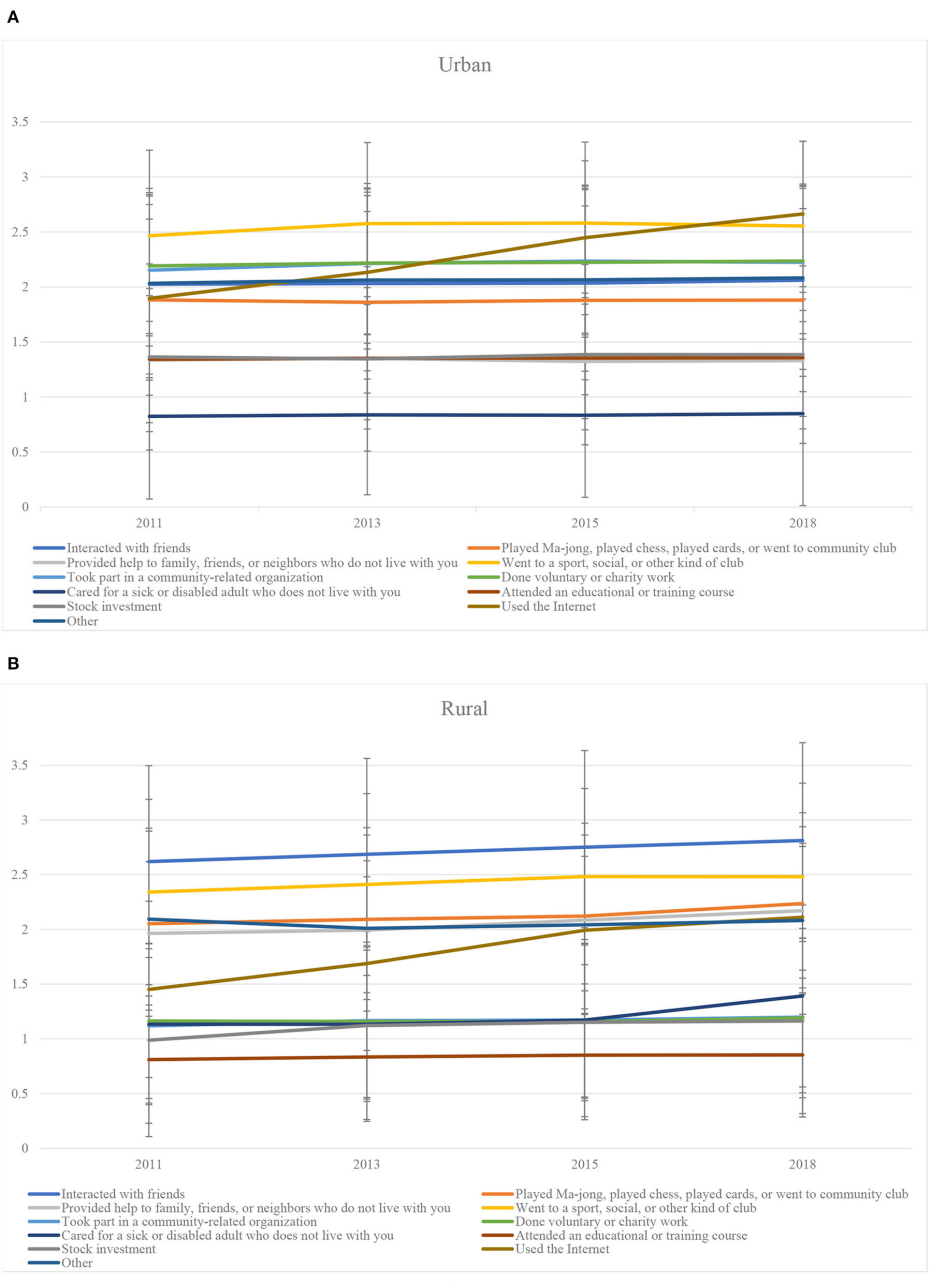
Distribution of social engagement activities.

As Figure 1 shows, rural respondents were more likely to participate in activities rooted in acquaintance networks (e.g., interacting or playing with and helping family, relatives, and friends), while urban respondents participated in activities organized by social organizations (e.g., voluntary or charity work and educational courses) or surfed the Internet.

As for the control variables, the mean value of monthly family per capita income of the urban respondents was nearly twice that of the rural respondents, who also had lower education, were more likely to have good self-care ability, and were less likely to suffer from other chronic diseases.

### Regression Analysis and Fairlie Decomposition Results

Table 3 shows the results of urban–rural disparity in self-management behaviors and the effect of social engagement thereof based on the benchmark random-effects panel logit regression models (Model 1). After controlling for confounding factors, we found that the urban respondents were less likely to cultivate tobacco avoidance behavior (OR = 0.833, *p* < 0.01). However, they were more like to use medication (45.6%) than rural respondents (OR = 1.456, *p* < 0.01). Social engagement had a significant, positive effect on all self-management behaviors. With each additional unit of social engagement, the probability of the respondents adhering to alcohol avoidance, tobacco avoidance, medication use, physical activity, and self-monitoring behaviors will increase by 5.2% (OR = 1.052, *p* < 0.01), 4.8% (OR = 1.048, *p* < 0.01), 8.5% (OR = 1.085, *p* < 0.01), 82.4% (OR = 1.824, *p* < 0.01), and 4.4% (OR = 1.044, *p* < 0.01), respectively.

**Table 3:** Random-effect panel logit regressions reporting urban-rural disparities of self-management behaviors.

**Model 1**	**Alcohol avoidance**	**Tobacco avoidance**	**Medication use**	**Physical activity**	**Self-monitoring**
Hukou (urban)	−0.122	−0.183^**^	0.376^**^	0.333	0.045
	(0.050)	(0.004)	(0.002)	(0.213)	(0.577)
Social engagement	0.051^***^	0.047^***^	0.082^***^	0.601^***^	0.043^***^
	(0.00)	(0.00)	(0.00)	(0.00)	(0.00)
Residence: urban	0.06	−0.072	0.219^*^	−0.387	−0.113
	(0.338)	(0.275)	(0.069)	(0.146)	(0.168)
Gender: female	−0.686^***^	−1.005^***^	0.345^***^	0.026	0.196^**^
	(0.00)	(0.00)	(0.00)	(0.904)	(0.001)
Age	−0.443^**^	−0.212	1.989^***^	−2.68^***^	0.35
	(0.002)	(0.148)	(0.00)	(0.00)	(0.06)
Spouse: yes	−0.055	−0.035	−0.023	0.222	0.109
	(0.355)	(0.564)	(0.841)	(0.383)	(0.15)
Education: junior school and above	0.315^***^	0.1	0.225^**^	−0.111	0.275^***^
	(0.00)	(0.051)	(0.008)	(0.525)	(0.00)
Basic medical insurance: yes	0.06	−0.292^*^	1.008^***^	−0.009	0.844^***^
	(0.599)	(0.01)	(0.00)	(0.98)	(0.00)
Self-care ability	−0.005	−0.004	0.029^*^	0.063^*^	0.007
	(0.517)	(0.573)	(0.023)	(0.015)	(0.467)
Multimorbidity: yes	0.005	0.038	0.319^***^	0.002	0.215^**^
	(0.918)	(0.478)	(0.00)	(0.992)	(0.001)
Absolute household income	−0.081^***^	−0.061^**^	−0.028	0.069	0.042
	(0.00)	(0.001)	(0.404)	(0.242)	(0.059)
2013	−0.079	0.567^***^	−0.508^***^	−1.804^***^	−1.549^***^
	(0.198)	(0.00)	(0.00)	(0.00)	(0.00)
2015	1.833^***^	2.485^***^	1.775^***^	0.188	−0.939^***^
	(0.00)	(0.00)	(0.00)	(0.212)	(0.00)
2018	0.417^***^	0.106	–.745^***^	11.838^***^	−1.078^***^
	(0.00)	(0.114)	(0.00)	(0.00)	(0.00)
Constant	1.33^*^	0.77	−8.273^***^	3.444	−0.969
	(0.035)	(0.24)	(0.00)	(0.239)	(0.24)
lnsig2u	−17.295	−17.295	1.299	4.461	−0.105

*** *p < 0.001*,

** *p < 0.01*,

* *p < 0.05*.

To understand the effect of social engagement on urban–rural disparity in the self-management behaviors of middle-aged and older hypertensive patients, we added the “hukou (urban)^*^social engagement” variable into Model 2 based on Model 1. As Table 4 shows, this variable had significant effects on middle-aged and older respondents' tobacco avoidance (β = −0.023, *p* < 0.05) and medication use (β = −0.077, *p* < 0.001) behaviors, indicating a significant negative effect of social engagement on the disparity in both behaviors between urban patients and rural respondents. Specifically, social engagement significantly increased the urban–rural disparity in their tobacco avoidance behavior (β = −0.171, *p* < 0.01), but significantly decreased the disparity in their medication use behavior (β = 0.375, *p* < 0.01). The Fairlie decomposition results in Table 5 reveal that the effect of social engagement was significant in tobacco avoidance and medication use behaviors, explaining 75.000% and 29.412% of the disparity, respectively.

**Table 4:** Random-effect panel logit regressions reporting the effect of social engagement on urban-rural disparities of self-management behaviors.

**Model 2**	**Alcohol avoidance**	**Tobacco avoidance**	**Medication use**	**Physical activity**	**Self-monitoring**
Hukou: urban	−0.118	−0.171^**^	0.375^**^	0.232	0.044
	(0.056)	(0.008)	(0.002)	(0.399)	(0.58)
Social engagement	0.051^***^	0.049^***^	0.086^***^	0.599^***^	0.042^***^
	(0.00)	(0.00)	(0.00)	(0.00)	(0.00)
Residence: urban	0.062	−0.066	0.236	−0.399	−0.115
	(0.327)	(0.314)	(0.051)	(0.131)	(0.161)
Gender: female	−0.685^***^	−1^***^	0.357^***^	0.042	0.194^**^
	(0.00)	(0.00)	(0.00)	(0.844)	(0.001)
Age	−0.441^**^	−0.208	2.006^***^	−2.44^***^	0.348
	(0.002)	(0.157)	(0.00)	(0.00)	(0.061)
Spouse: yes	−0.055	−0.036	−0.025	0.227	0.109
	(0.353)	(0.556)	(0.83)	(0.368)	(0.149)
Education: junior school and above	0.315^***^	0.102^*^	0.228^**^	−0.106	0.275^***^
	(0.00)	(0.048)	(0.007)	(0.539)	(0.00)
Medical insurance: yes	0.059	−0.295^**^	1.004^***^	0.006	0.844^***^
	(0.603)	(0.009)	(0.00)	(0.986)	(0.00)
Self-care ability	−0.005	−0.005	0.028^*^	0.061^*^	0.007
	(0.506)	(0.53)	(0.028)	(0.017)	(0.455)
Multimorbidity: yes	0.005	0.036	0.314^***^	0.006	0.215^**^
	(0.926)	(0.497)	(0.00)	(0.975)	(0.001)
Absolute household income	−0.08^***^	−0.06^***^	−0.025	0.07	0.042
	(0.00)	(0.002)	(0.461)	(0.23)	(0.062)
Hukou: urban × Social engagement	−0.006	−0.023^*^	−0.077^***^	−0.159^**^	0.011
	(0.459)	(0.01)	(0.00)	(0.001)	(0.355)
2013	−0.079	0.567^***^	−0.507^***^	−1.79^***^	−1.548^***^
	(0.198)	(0.00)	(0.00)	(0.00)	(0.00)
2015	1.833^***^	2.486^***^	1.775^***^	0.179	−0.939^***^
	(0.00)	(0.00)	(0.00)	(0.229)	(0.00)
2018	0.417^***^	0.107	−0.741^***^	11.631^***^	−1.079^***^
	(0.00)	(0.11)	(0.00)	(0.00)	(0.00)
Constant	1.322^*^	0.743	−8.349^***^	2.624	−0.956
	(0.037)	(0.257)	(0.00)	(0.365)	(0.246)
lnsig2u	−17.295	−17.293	1.296	4.418	−0.109

*** *p < 0.001*,

** *p < 0.01*,

* *p < 0.05*.

**Table 5:** Fairlie decomposition results of the effect of social engagement on the urban-rural disparity in tobacco avoidance and medication use behaviors among older adults.

	**Tobacco avoidance**			**Medication use**		
	**β**	**Robust standard error**	**Contribution (%)**	**β**	**Robust standard error**	**Contribution (%)**
Rural	0.465^**^	0.005		0.787^**^	0.004	
Urban	0.442^**^	0.009		0.841^**^	0.006	
Difference	0.024^*^	0.010		−0.055^**^	0.008	
Explained: Difference due to endowments effect	−0.016	0.013	−66.667	−0.004	0.010	30.909
Social engagement	−0.012^**^	0.003	75.000	−0.005^**^	0.002	29.412
Unexplained: Difference due to coefficients effect	0.039	0.016	162.500	−0.051	0.012	69.091

** *p < 0.01*,

* *p < 0.05*.

To determine the effect of population mobility on the relationship of social engagement and urban–rural disparity in self-management behaviors, we introduced “hukou (urban)^*^social engagement^*^residence” into Model 3 based on Model 2. The results (Table 6) show that population mobility significantly weakened the role of social engagement in narrowing urban–rural disparity for medication use behavior (β = −0.115, *p* < 0.05). We further analyzed the effect of population mobility on the relationship between social engagement and medication use behavior among urban and rural respondents, respectively. The results show that the weakening effect of population mobility reflected the flow of urban population to rural areas (β = −0.157, *p* < 0.01) (Table 7).

**Table 6:** Random-effect panel logit regressions reporting the role of population mobility for the effect of social engagement on urban-rural disparities of self-management behaviors.

**Model 3**	**Alcohol avoidance**	**Tobacco avoidance**	**Medication use**	**Physical activity**	**Self-monitoring**
Hukou: urban	−0.118	−0.171^**^	0.382^**^	0.234	0.044
	(0.057)	(0.008)	(0.002)	(0.395)	(0.582)
Social participation	0.05^***^	0.049^***^	0.1^***^	0.592^***^	0.041^***^
	(0.00)	(0.00)	(0.00)	(0.00)	(0.00)
Residence: urban	0.059	−0.067	0.263^**^	−0.409	−0.116
	(0.351)	(0.31)	(0.031)	(0.121)	(0.158)
Gender: female	−0.685^***^	−1^***^	0.356^***^	0.042	0.194^**^
	(0.00)	(0.00)	(0.00)	(0.841)	(0.001)
Age	−0.441^**^	−0.208	2.007^***^	−2.434^***^	0.348
	(0.002)	(0.157)	(0.00)	(0.00)	(0.061)
Spouse: yes	−0.055	−0.036	−0.026	0.228	0.109
	(0.354)	(0.557)	(0.822)	(0.365)	(0.149)
Education: junior school and above	0.315^***^	0.102^*^	0.228^**^	−0.108	0.275^***^
	(0.00)	(0.048)	(0.007)	(0.533)	(0.00)
Medical insurance: yes	0.059	−0.295^**^	1.006^***^	0.011	0.844^***^
	(0.606)	(0.009)	(0.00)	(0.974)	(0.00)
Self-care ability	−0.005	−0.005	0.028^*^	0.061^*^	0.007
	(0.506)	(0.531)	(0.028)	(0.017)	(0.454)
Multimorbidity: yes	0.005	0.036	0.315^***^	0.006	0.215^**^
	(0.927)	(0.497)	(0.00)	(0.975)	(0.001)
Absolute household income	−0.08^***^	−0.06^**^	−0.026	0.07	0.042
	(0.00)	(0.002)	(0.447)	(0.228)	(0.062)
Hukou: urban × Social engagement	−0.009	−0.024^*^	−0.041	−0.173^**^	0.009
	(0.405)	(0.034)	(0.089)	(0.002)	(0.54)
Hukou: urban × Social engagement × Residence: urban	0.008	0.002	−0.115^*^	0.046	0.007
	(0.689)	(0.908)	(0.01)	(0.599)	(0.816)
2013	−0.078	0.568^***^	−0.509^***^	−1.789^***^	−1.548^***^
	(0.201)	(0.00)	(0.00)	(0.00)	(0.00)
2015	1.833^***^	2.486^***^	1.775^***^	0.181	−0.939^***^
	(0.00)	(0.00)	(0.00)	(0.225)	(0.00)
2018	0.418^***^	0.107	−0.743^***^	11.623^***^	−1.078^***^
	(0.00)	(0.109)	(0.00)	(0.00)	(0.00)
Constant	1.327^*^	0.744	−8.42^***^	2.626	−0.953
	(0.036)	(0.256)	(0.00)	(0.364)	(0.247)
lnsig2u	−17.294	−17.294	1.297	4.417	−0.109

*** *p < 0.001*,

** *p < 0.01*,

* *p < 0.05*.

**Table 7:** Random-effect panel logit regressions reporting the effect of population mobility on social engagement among urban and rural patients in medication use behavior.

	**Medication use (rural)**	**Medication use (urban)**
Social engagement	0.094^***^	0.055^*^
	(0.00)	(0.037)
Residence: urban	0.185	0.335
	(0.227)	(0.228)
Gender: female	0.375^***^	0.213
	(0.00)	(0.451)
Age	1.528^***^	4.779^***^
	(0.00)	(0.00)
Spouse: yes	0.001	−0.228
	(0.997)	(0.556)
Education: junior school and above	0.214^*^	0.047
	(0.014)	(0.873)
Basic medical insurance: yes	1.195^***^	−0.102
	(0.00)	(0.848)
Self-care ability	0.025	0.036
	(0.067)	(0.364)
Multimorbidity: yes	0.275^**^	0.54
	(0.002)	(0.062)
Absolute household income	−0.004	−0.171
	(0.921)	(0.092)
Social engagement × Residence: urban	−0.005	−0.157^**^
	(0.883)	(0.005)
2013	−0.404^***^	−1.228^***^
	(0.00)	(0.00)
2015	1.768^***^	2.146^***^
	(0.00)	(0.00)
2018	−0.688^***^	−1.259^***^
	(0.00)	(0.00)
Constant	−6.9^***^	−15.245^***^
	(0.00)	(0.00)
lnsig2u	0.886	2.775

*** *p < 0.01*,

** *p < 0.05*,

* *p < 0.1*.

## Discussion

Self-management of long-term condition is the core of chronic disease management. It critically supplements limited formal healthcare networks in order to manage the demand for health services. It has become crucial to facilitate healthy aging through self-management of chronic illness, especially given population aging, the increased economic and health burden of chronic disease, urban–rural dualities in social resources and culture, and flow of migration between urban and rural areas in China. We analyzed the data from 2011, 2013, 2015, and 2018 waves of the China Health and Retirement Longitudinal Study (CHARLS) and found that: first, medication use behavior among urban middle-aged and older patients was significantly better, whereas tobacco avoidance behavior was significantly lower compared with the rural population; second, ~75.000% and 29.412% of the explained urban–rural gap in tobacco avoidance and medication use, respectively, could be attributed to social engagement; third, the negative effect of social engagement on urban–rural disparity in medication use increased when urban residents moved to rural areas.

In the urban-rural disparity in self-management behaviors of middle-aged and older patients with hypertension disorders, we found that self-management behaviors among urban residents were not always better than that of their rural counterparts. While urban residents showed better medication use (44–46), they were less likely to avoid tobacco and alcohol use. We attribute these differences to China's household registration system that was formally established in 1958, that is, the urban–rural segmentation has led to unfair distributions of resources (e.g., healthcare and cultural or educational resources) and different social relationships as well (4, 5). While urban residents may benefit from better access to social welfare, healthcare, institutional social support, and medical information (4, 47), rural residents benefit more from stronger social cohesion and close acquaintance networks. Indeed, close family supervision and stronger peer effect may help rural residents in avoiding tobacco and alcohol (48). Our results verify this interpretation of the urban–rural disparity even if, overall, social engagement is a positive factor for individual's self-management behaviors.

While social engagement enlarged the urban–rural gap in tobacco avoidance behavior, it narrowed it for medication use. Indeed, the Fairlie decomposition revealed that ~75.000% and 29.412% of the urban–rural gap in tobacco avoidance and medication use, respectively, could be attributed to differences in social engagement. Further analysis revealed that rural patients were more likely to participate in activities based on acquaintance networks, while urban patients often participated in activities organized by social organizations or surfed the Internet. We, thus, confirm that social engagement in rural China is still dominated by traditional, stable, and strong kinship within the same village. Putnam's (2000) social capital theory (49) specifically states that people who have more social engagement also create closer and more stable social ties and have more trust in others, which is useful for communal integration and harmony (22). Thus, “reminders,” “persuasion,” and “encouragement” against tobacco use likely benefit rural patients' self-management of chronic illness. However, it should be noted that the same population is constrained by limited health literacy and poor access to healthcare resources (e.g., primary care providers, specialist doctors, and online healthcare information) (14, 50, 51). Thus, middle-aged and older rural patients may not fully benefit from their social networks when it comes to medication use behavior (19, 52). On the contrary, social engagement in urban was characterized by broader opportunities for participation in social organizations as well as greater mobility (46). Although this social relationship may be not conducive to adherence to tobacco avoidance behavior, more developed public healthcare services and voluntary or informal organizations may help patients make right choices and learn to adopt healthy behaviors, such as medication use (13, 25, 26, 53). However, the “urban health penalty” may also expose residents to higher stress, worsening their tobacco use (54).

There was an insignificant urban–rural disparity in self-monitoring, physical activity, and alcohol avoidance behaviors among urban and rural respondents, which may reflect differences in social engagement and accordingly play a role in self-management behaviors. The reduced disparity may also be the result of effective welfare policy (e.g., the community-based hypertension management service of China's Equalization of Basic Public Health Services policy in 2009, new urbanization in 2014, and the rural revitalization strategy in 2017). Urban areas today have more voluntary or informal organizations than ever before to increase residents' participation in positive and beneficial social activities (5), including volunteering, to better connect with their community (2). Consequently, Chinese middle-aged and older urban residents tend to have more diverse and higher rates of social engagements than their rural counterparts (6); the influence of this resource to help patients adhere to self-monitoring, physical activity, and alcohol avoidance works in a similar fashion to the influence of close kinship and acquaintance networks in rural areas (2). China's Equalization of Basic Public Health Services has also helped narrow the urban–rural gap in basic screening, follow-up evaluation, and health education. The new-type urbanization in 2014 and rural revitalization strategy in 2017 have increased flow of labor between rural and urban areas in both directions. These trends have had the effect of expanding overall access to social welfare, and thus narrow gaps in self-management knowledge, ability, and behaviors among hypertension sufferers, especially pertaining to common risk factors (e.g., lack of exercise, overweight/obesity, and alcohol consumption) (55).

Finally, the narrowing effect of social engagement on urban–rural disparity in medication use increased when urban residents moved to rural areas. Given China's geographical size, and the long migration patterns and urban/rural transformations, traditional urban–rural divisions are starting to blur. Most public services and welfare benefits (e.g., education, healthcare, and social security) are no longer entirely attached to a person's hukou status, but also related to their physical location (31). However, the narrowing effect for medication use has not improved the effect of social engagement on such behavior among rural residents, but decreased more in urban areas. Thus, while the household registration system is less rigid, gap remain in the access to public healthcare services (28, 30).

There are some limitations to our study that must be acknowledged. First, there were deficiencies in types and assessments of self-management behaviors included in the study; for instance, sleep, psychological management, and eating behaviors were not analyzed. The social engagement measures included herein are not exhaustive, and should not be treated as such; for instance, other forms of informal contact, such as telephoning or home visits, were not assessed. Second, CHARLS is a pooled cross-sectional dataset, which may contribute to a sample bias. Finally, our study only included the result of four waves. Longer longitudinal studies should be conducted when such data become available.

## Conclusions

Hypertension is highly prevalent across China. Despite reforms and policies to reduce the urban–rural gap in social welfare and public healthcare services for patients, common self-management behaviors, such as self-monitoring, physical activity, and alcohol avoidance, have not revealed significant differences between urban and rural middle-aged and older patients. We show that social engagement is crucial to reduce urban–rural differences self-management. The Chinese government should, thus, commit more strongly to eliminating the negative effects of the hukou institution and population mobility in order to reduce differences in social engagement among rural and urban residents.

First, it must continue to leverage “soft” public services to reduce the urban–rural disparity; take maximum advantage of the relative and acquaintances effect on rural areas and professional guidance available in urban areas; as well as organize family, peer, and community engagement groups to supervise, assistant, and teach patients correct self-management. Second, social organizations should be tasked with compensating for inadequacies in organic social engagement in rural areas. The government should encourage professionals to join local non-government organizations to promote health information, both online and offline, and conduct easy lectures and trainings. Third, to reduce the deepening negative effect of social engagement on tobacco avoidance behavior in urban areas as well as the effect of “smoking culture,” group intervention programs should be established for patients.

## Data Availability Statement

Publicly available datasets were analyzed in this study. This data can be found at: http://charls.pku.edu.cn/.

## Author Contributions

JL designed the study and drafted the manuscript. LL and YW analyzed the data. JL and ZZ obtained funding. ZZ supervised the study. All authors have read and approved the final manuscript.

## Funding

This study was funded by National Natural Science Foundation of China (Project Approval Nos. 71804101 and 71874137).

## Conflict of Interest

The authors declare that the research was conducted in the absence of any commercial or financial relationships that could be construed as a potential conflict of interest.

## Publisher's Note

All claims expressed in this article are solely those of the authors and do not necessarily represent those of their affiliated organizations, or those of the publisher, the editors and the reviewers. Any product that may be evaluated in this article, or claim that may be made by its manufacturer, is not guaranteed or endorsed by the publisher.

## Footnotes

^1^That is, interacted with friend; played ma-jong, chess, or cards, or went to a community club; provided help to family, friends, or neighbors who do not live with you and did not pay you for the help; went to a sport, social, or other kind of club; took part in a community organization; did voluntary or charity work; cared for a sick or disabled adult who does not live with you and who did not pay you for the help; attended an educational or training course; engaged in stock investment; used the Internet; other social activities.

^2^Information about chronic diseases was obtained by asking participants “Have you been told by doctors or do you know that you have at least one of the following 14 diseases: hypertension, dyslipidemia, diabetes/high blood, cancer or other malignant tumors, chronic lung disease, liver disease, heart disease, stroke, kidney disease, stomach disease/digestive system diseases, emotional and spiritual diseases, memories related diseases, arthritis/rheumatism, or asthma?”.
